# Characterization of *Schistosoma japonicum* tetraspanning orphan receptor and its role in binding to complement C2 and immunoprotection against murine schistosomiasis

**DOI:** 10.1186/s13071-017-2229-y

**Published:** 2017-06-09

**Authors:** Shuai Ma, Jinli Zai, Yanhui Han, Yang Hong, Min Zhang, Xiaodan Cao, Qian Han, Ke Lu, Zhixin Zhao, Jiaojiao Lin, Zhiqiang Fu

**Affiliations:** 1 Key Laboratory of Animal Parasitology, Ministry of Agriculture, Shanghai Veterinary Research Institute, Chinese Academy of Agricultural Sciences, Shanghai, 200241 China; 2 College of Animal Science, Henan Institute of Science and Technology, Xinxiang, Henan Province 453003 China; 3 College of Animal Science and Technology, Henan University of Science and Technology, Luoyang, Henan Province 471023 China; 4 Jiangsu Co-innovation Center for Prevention and Control of Important Animal Infectious Diseases and Zoonoses, Yangzhou, 225009 China

**Keywords:** *Schistosoma japonicum*, Tetraspanning orphan receptor, C2-binding receptor, Vaccine

## Abstract

**Background:**

Schistosomiasis remains an important global public health problem, as millions of people are at risk of acquiring infection. An ideal method for sustainable control of schistosomiasis would be to develop an efficient vaccine. Schistosomes can survive in the host vascular system by immune evasion, regulating the host complement cascade. *Schistosoma japonicum* tetraspanning orphan receptor (SjTOR) is a complement regulator, which is a tegument membrane protein. To date there is no experimental evidence to explain the function of SjTOR.

**Results:**

We cloned the first extracellular domain of the SjTOR (SjTOR-ed1) gene and expressed the gene in *Escherichia coli.* The expression level of SjTOR in different developmental stages of *S. japonicum* was assessed by quantitative real-time RT-PCR. Western blotting showed that recombinant SjTOR-ed1 (rSjTOR-ed1) could be recognised by schistosome-infected mouse serum. Immunolocalization indicated that the protein was mainly distributed on the tegument of the parasite. Haemolytic assays and ELISA revealed that rSjTOR-ed1 could inhibit complement hemolysis and bind to complement C2. Purified rSjTOR-ed1 emulsified with ISA206 adjuvant could induce a significant reduction of worm burden from 24.51 to 26.51%, and liver egg numbers from 32.92 to 39.62% in two independent trials in mice.

**Conclusions:**

The results of this study indicated that rSjTOR-ed1 could inhibit complement hemolysis and bind to complement C2, and it is a potential vaccine candidate that protects against *S. japonicum* infection.

## Background

Schistosomiasis is a neglected tropical disease in humans, and it remains a serious global public health problem that affects over 200 million people in 74 developing countries and causes hundreds of thousands of deaths annually [1, 2]. In China, nearly 185,000 people are currently infected with *Schistosoma japonicum* [3], and another 68 million individuals are at risk of infection [4]. Most human cases (> 82%) occur in the lake and marshland regions of southern China [5]. In addition to humans, more than 40 kinds of livestock and wild animals are reservoir hosts for *S. japonicum*, and bovines are the major source of infection, especially in the lake/marsh endemic area of the Yangtze River. Although treatments have been available for several decades, schistosomiasis remains an important public health concern in China. As snail control is always difficult to achieve [6], and treatment with praziquantel does not prevent a high frequency of reinfection [7], schistosomiasis is difficult to control. Thus, to control this disease, there is an urgent need to develop alternative therapies and effective vaccines [8] that could reduce the release of eggs and restrict parasite transmission or induce which kind of activity against schistosome infection.

Schistosomes can survive in the inhospitable environment of the bloodstream that contains many different immunological effectors [9], such as host complement, which is an important effector of vertebrate host defence against the invading parasite [10]. Schistosomes must evade the host immune responses, which is attributed in part to their tegument [11]. Thus, schistosomal proteins on the surface of the tegument and some regulatory proteins that impede the complement cascade might represent ideal molecules for the discovery of vaccine candidates [9, 12–14].

Trispanning orphan receptor represents a novel family of transmembrane receptors, the first member of which was found in *S. haematobium*, and then in *S. mansoni* and *Trypanosoma cruzi* [15, 16]. Tetraspanning orphan receptor (TOR) is a new name for trispanning orphan receptor after finding a fourth transmembrane domain of the molecule in *S. mansoni* [10]. In *S. haematobium* and *S. mansoni*, TOR was found to be a transmembrane protein located on the tegumental surface of adult worms [10] and was a complement regulatory receptor that targeted the early complement pathway [15]. Because it shares antigenic determinants with the C2-binding complement component C4 and can act as a decoy C2-binding receptor, TOR can regulate complement activation by interfering with the formation of C3 convertase (C4b2a) [9, 15, 17]. Further experiments indicated that a short amino acid sequence of the ShTOR extracellular domain 1 (ShTOR-ed1) containing the C-terminal H17 motif binds C2 and interfered with its cleavage by C1s, thereby limiting complement C3 convertase formation [13, 18, 19]. Additionally, the highest expression level in cercariae and its surface localisation in *S. mansoni* not only indicates that TOR is a complement regulator in the early period of infection but also represents an interesting vaccine candidate [13]. TOR in *S. japonicum* was first found by Liu et al. [20] in cercariae, schistosomula, and adult worms. TOR was also identified in a proteomics study of tegument surface proteins of *S. japonicum* in our laboratory [21], although no further studies on SjTOR have yet been reported.

Herein, we cloned and expressed the SjTOR-ed1 gene, detected the immunogenicity of rSjTOR-ed1 that contained the C2 binding region, and analysed the expression levels of the SjTOR gene among different developmental stages of *S. japonicum* and the localisation of the protein. We also investigated the complement regulatory function of rSjTOR-ed1 by haemolytic assays and ELISA and evaluated its protective efficacy against schistosome infection induced by rSjTOR-ed1 protein in a murine model.

## Methods

### Parasites and animals

The life-cycle of *S. japonicum* (Chinese mainland strain, Anhui isolate) were maintained in New Zealand rabbits and *Oncomelania hupensis* snails at the Shanghai Veterinary Research Institute, Chinese Academy of Agricultural Sciences. Schistosomes of 3, 7, 14, 21, 28, 35 and 42 days were collected by perfusion of New Zealand rabbits that were artificially infected with *S. japonicum* cercariae [22–24]. Adult worms (42 days) were separated into males and females manually. Cercariae were collected from schistosome-infected snails, and eggs were isolated from the livers of infected mice using previously described methods [8, 25]. Newly transformed (3 h) and skin-stage (24 h) schistosomes were developed from cercariae according to Basch’s method, briefly cercariae were sheared through a double-Luer-ended needle connected to two syringes, and tails separated and discarded [26]. Male 6-week-old BALB/c mice were purchased from Shanghai Laboratory Animal Center, Chinese Academy of Sciences (Shanghai).

### Bioinformatics analysis of SjTOR

The protein sequence of SjTOR (Q5DC12) was obtained by searching against the UniProt database (http://www.uniprot.org/) using TOR_SCHJA as a query. Signal peptide prediction was performed using SignalP (http://www.cbs.dtu.dk/services/SignalP/) and N-glycosylation sites were analyzed using NetNGlyc 1.0 (www.cbs.dtu.dk/services/NetNGlyc/). Prediction of transmembrane helices was carried out using the TMHMM Server version 2.0 (http://www.cbs.dtu.dk/services/TMHMM-2.0/). Secondary structure prediction analysis was performed using PSIPRED (http://bioinf.cs.ucl.ac.uk/psipred/). Multiple amino acid (AA) sequence alignments were generated using ClustalX software (http://www.clustal.org/).

### Real-time RT-PCR analysis of SjTOR expression in different developmental stages of schistosomes

Total RNA was extracted from *S. japonicum* at different stages using TRIzol® Reagent (Invitrogen, Carlsbad, USA), as per the manufacturer’s instructions. Then, cDNA was synthesised according to standard protocols using PrimeScript RT Reagent Kit with gDNA Eraser (TaKaRa, Shiga, Japan). Primers for real-time PCR were designed using the primer design tool Beacon Designer 7.0. The SjTOR primers (forward: 5′-AGC CTA CTG TCT TGG TAT GGT GTG-3′; reverse: 5′-AGC CCT TGT GTT AGA CTC GTT GG-3′) amplified a product of 196 bp. The primers targeting the *S. japonicum* α-tubulin gene (forward: 5′-CTG ATT TTC CAT TCG TTT G-3′; reverse: 5′-GTT GTC TAC CAT GTT GGC A-3′) amplified a product of 213 bp, which was used as an internal standard. Real-time PCR was performed with SYBR Premix Ex Taq™ kit (TaKaRa) in a Mastercycler Ep Reaplex (Eppendorf, Hamburg, Germany). No template controls were included in each PCR run. The analysis was repeated three times.

### Expression and purification of recombinant protein

Primers were designed based on the predicted first extracellular domain (AA 49–167) and the nucleotide sequences of the SjTOR open reading frame (GenBank AY814912.1). The first extracellular domain (AA 49–167) was amplified using the forward and reverse oligonucleotides as primers, 5′-GCG GAA TTC ATG ACG TTT AAT CCG-3′ and 5′-CGC CTC GAG TCA AAT GTA TGG ACT-3′ (*EcoR*I and *Xho*I sites underlined) Then, the PCR-generated fragment that encoded the first extracellular domain of SjTOR was inserted into the expression vector pET28a(+) (Novagen, Darmstadt, Germany). The resultant recombinant plasmid pET28a(+)–SjTOR-ed1 was transformed into *Escherichia coli* BL21 (*DE3*) cells (Invitrogen). For protein expression, the transformed cells were grown in LB medium, and the gene was overexpressed in BL21 (*DE3*) cells using 1 mM isopropyl-β-D-thiogalactopyranoside (IPTG) at 37 °C for 5–6 h. Cells were collected by centrifugation at 4 °C and centrifuged at 12000× *rpm* for 20 min. Cell pellets were suspended in PBS (pH 7.4), lysed by sonication and pelleted by centrifugation. The pellets were resuspended in 5 ml PBS with 8 M urea. Then the crude extract was analysed by SDS-PAGE. The recombinant protein rSjTOR-ed1 was purified by Ni-NTA affinity chromatography (Novagen, Darmstadt, Germany) and dialysed against PBS (pH 7.4), containing decreasing concentrations of urea (6, 4, 3, 2, 1 and 0 M). Purified rSjTOR-ed1 was used to immunise BALB/c mice by subcutaneous injections (20 μg at each injection) to collect polyclonal antibodies, which were used for the following Western blot and immunolocalization assays.

### Western blot analysis

Purified rSjTOR-ed1 protein was subjected to SDS-PAGE and then electroblotted onto a NC membrane (Whatman, Munich, Germany) at 135 mA for 70 min at 4 °C. Membranes were blocked with PBST plus 5% (*w*/*v*) nonfat milk for 1 h at 37 °C with constant shaking, and were probed with serum from mice infected with *S. japonicum*, anti-rSjTOR-ed1 mouse serum, or normal mouse serum diluted 1:100 in PBST overnight at 4 °C. After washing, membranes were probed with goat anti-mouse IgG conjugated to horseradish peroxidase (HRP) diluted 1:3000 in PBST for 1 h at room temperature (RT). Finally, the immunoreactive bands were visualised using DAB Substrate Solution (Tiangen Biotech, Beijing, China) after three washes with PBST.

### Immunolocalization

To analyse the tissue expression of SjTOR, the frozen sections (8 μm thick, 35-day worms), cercariae and the paraffin embedded sections (5 μm thick, 35-day worms and livers from infected BALB/c mice) were prepared and fixed. Then the indirect immunofluorescence assays and the immunohistochemistry assay were performed as follows respectively.

The frozen sections were blocked with normal goat serum (Yeasen, Beijing, China) for 2 h at RT and incubated with anti-rSjTOR-ed1 serum (1:100 dilution) overnight at 4 °C. Meanwhile naive mice serum was used as a negative control. Samples were probed with CY3-conjugated goat anti-mouse IgG (Rockland, USA), which was diluted 1:2000 and incubated for 1 h at RT. Intervening washes with PBST were conducted after incubation when necessary. Then sections were stained with 2-(4-amidinophenyl)-6-indolecarbamidine dihydrochloride (DAPI) at a final concentration of 10 μg/ml for 8 min at RT. Parasites were observed by fluorescence microscopy (Nikon, Tokyo, Japan).

The cercariae were collected and fixed in 80% (*v*/v) ethanol in PBS (pH 7.4). Then they were blocked with normal swine serum (Yeasen, Beijing, China) for 1 h at RT and treated as described above except being redyed with DAPI. The cercariae were then put on the micro-slide and visualised by the confocal laser scanning microscopy (Zeiss, Baden Wurttemberg, Germany).

The paraffin-embedded sections were deparaffinized in xylene, hydrated with 75% ethanol, and quenched with 3% (*w*/*v*) H_2_O_2_ in PBS (pH 7.4) to remove the endogenous peroxidase. To expose the antigens, sections were boiled in citrate buffer (pH 6.0) for 10 min [27]. The sections were then blocked with normal goat serum for 2 h and incubated in anti-rSjTOR-ed1 serum (1:100 dilution) overnight at 4 °C. After washing three times with PBST, the slides were incubated in biotinylated second antibody at RT for 1 h and subsequently stained with DAB (DAKO, Glostrup, Denmark). Finally, the sections were visualised by microscopy after they were counterstained with hematoxylin and mounted with neutral balsam.

### Haemolytic assays

Inhibition of complement activation was determined based on a modified method of Inal et al. [28] as follows. Briefly, different concentration ranges (0.01 to ~10 μM) of rSjTOR-ed1 protein and Albumin Bovine V (Sigma-Aldrich, Saint Louis, USA) in 50 μl freshly prepared gelatin veronal buffer (GVB^2+^, pH 7.4) were pre-incubated with 100 μl normal guinea pig serum (NGpS, diluted 1/340 in GVB^2+^) for 30 min at room temperature, then was added to 50 μl sheep erythrocytes (EA, 2 × 10^8^ cell/ml, coated with antibodies). The mixtures were incubated at 37 °C for 30 min and then centrifuged (6000× *g*, 2 min). The controls, both made up to 200 μl with GVB^2+^ consisting of cells alone (blank control) or cells with 100 μl diluted NGpS (positive control). A 100% lysis control (lysed by water) was executed as surveillance. Hemolysis was determined by measuring the absorbance of the supernatant at 412 nm. The anti-complement activity of samples was expressed as the percentage inhibition of hemolysis (compared with the positive control).

To further demonstrate the anti-complement activity of rSjTOR-ed1, we tested the effect of pre-challenge anti-SjTOR-ed1 serum to inhibit the action of rSjTOR-ed1 in the complement activation assay. The pre-challenge anti-SjTOR-ed1 serum (10 μl), normal mouse serum and freshly prepared gelatin veronal buffer (GVB^2+^, pH 7.4) were firstly used to neutralise the rSjTOR-ed1 protein (2 μM, in GVB^2+^) for 1 h at room temperature. Then the assay was performed as described above. The two assays were repeated three times each.

### ELISA

To detect the binding of C2 to rSjTOR-ed1, the same concentration of rSjTOR-ed1 and Albumin Bovine V (10 μg/ml) in carbonate-bicarbonate buffer (pH 9.6) was coated on a 96-well plate (Costar, Kennebunk, USA) overnight at 4 °C. The plate was washed with PBST and blocked with 0.5% gelatin (Amresco, Solon, USA) in PBST for 1 h at 37 °C. After washing, decreasing amounts of human complement C2 (Sino Biological Inc., Beijing, China) was diluted in 20 mM Tris buffer (containing 1 mM MgCl_2_ and 1 mM CaCl_2_) and incubated for 2 h at RT. Then, plates were washed and incubated with rabbit monoclonal antibody to C2 (Sino Biological Inc.) diluted 1:2000 in PBST for 70 min at 37 °C. After additional washes, plates were incubated with goat anti-rabbit IgG conjugated to HRP (Sigma-Aldrich), 1:4000 in PBST for 1 h at 37 °C. Plates were washed, and 3,3′5,5′-tetramethyl benzidine dihydrochloride was added as a substrate solution. Finally, the reaction was stopped with 50 μl 2 M H_2_SO_4_, and absorbance was measured at 450 nm.

### Evaluation of immune protective efficacy of rSjTOR-ed1

Six-week-old male BALB/c mice were divided randomly into three groups. Mice in rSjTOR-ed1 vaccination group were injected subcutaneously at 2-week intervals (on weeks 0, 2 and 4) with 20 μg rSjTOR-ed1 emulsified with ISA 206 (Seppic, Paris, France). While mice in the adjuvant control and blank control groups were immunised with ISA 206 in PBS or PBS only, respectively. Serum was collected by retro-orbital bleeding before the first vaccination and 1 week after each vaccination.

Two weeks after the final vaccination, mice were challenged percutaneously with 40 ± 1 viable cercariae, as described by Smithers and Terry [14, 29]. Six weeks after challenge, worms were perfused from the hepatic portal system and counted. To estimate the egg burden in the liver, the weight of the liver tissue was measured and homogenised in 15 ml PBS, and 2 ml homogenate was mixed with 2 ml 10% (*w*/*v*) NaOH and incubated at 56 °C for 1 h. The average of 3 counts per 50 μl mixture was taken as the number of eggs in each sample tested, then was converted into eggs per gramme (EPG). The assays were repeated in two independent trials. The rate of reduction in the worm and egg counts was calculated as follows:

Worm/egg reduction rate (%) = (numbers of worms/eggs in the ISA206 adjuvant group − numbers of worms/eggs in the rSjTOR-ed1 group)/numbers of worms/eggs in the ISA206 adjuvant group × 100% [30].

### Detection of specific antibodies

ELISA assay was performed to detect the specific anti-rSjTOR-ed1 antibodies. First, 96-well plates (Costar) were coated with 10 μg/mL rSjTOR-ed1 overnight at 4 °C and were blocked for 1 h at 37 °C with 1.5% BSA in PBST for 1 h at 37 °C. Then, 100 μl of each serum sample (1:100 dilution) was added and incubated for 2 h at RT. Plate-bound antibody was detected with peroxidase-conjugated anti-mouse IgG (Sigma-Aldrich), IgG1, and IgG2a (AbD Serotec, Kidlington, UK) that were each diluted in PBST to 1:3000. Finally, plates were developed as described previously.

### Statistical analysis

Data are expressed as means ± standard deviation (SD). Statistical analyses were performed with Student’s *t*-test or ANOVA using the Excel programme of Microsoft Office 2003.

## Results

### Sequence analysis of SjTOR

**Fig. 1:**
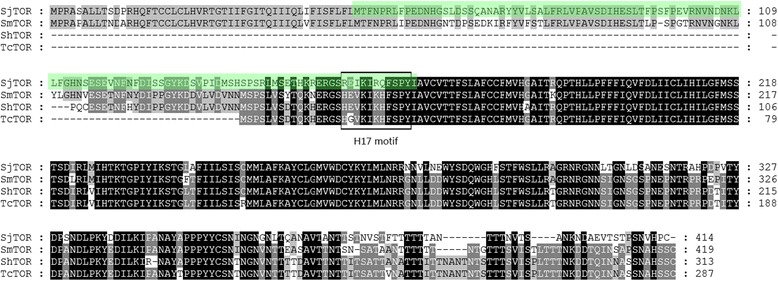
ClustalX alignment of the amino acid sequences of SjTOR (Q5DC12), SmTOR (C4QM85), ShTOR (Q9BLM6), and TcTOR (Q5J7P3). The peptide sequences shown to bind C2 are boxed. Regions with high identity and similarity among TOR sequences are indicated by *black* and *grey* columns. The first extracellular domain is shaded in *green* (AA 49–167)

The SjTOR protein consists of 414 amino acids and has a predicted molecular mass of ~46.5 kDa. The sequence analysis indicated that SjTOR did not possess a signal peptide, but did contain five N-glycosylation sites. Transmembrane domain and structure prediction analyses indicated that SjTOR contained four transmembrane domains. A comparison of amino acid sequences showed that SjTOR was 77.0, 72.0, and 75.0% identical to orthologs from *S. mansoni*, *S. haematobium*, and *T. cruzi*, respectively. The 11 amino acids (termed the H17 motif) in the C-terminal part of the first extracellular domain (ed1), which have been described to bind to C2 and interfere with its cleavage [10], are indicated by boxes (Fig. 1). The sequence of this domain of SjTOR shares a high level of sequence identity with other TORs, although there are four amino acid differences compared with the TORs of other *Schistosoma* species (Fig. 1).

### Transcription levels of SjTOR at different stages of the *S. japonicum* life-cycle

**Fig. 2:**
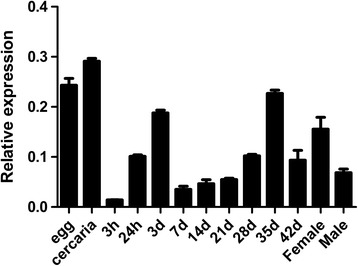
Analysis of SjTOR expression in different development stages. Samples throughout the 11 developmental stages of *S. japonicum* and between male and female adult worms were analysed by real-time PCR. Data were normalised against level of an internal housekeeping control gene (α-tubulin)

Levels of SjTOR transcript expression were investigated in *S. japonicum* eggs, cercariae, schistosomula, and adult worms as well as 42-day-old males and females using qRT-PCR analysis, with the *S. japonicum* tubulin gene as the housekeeping control gene. The result showed that SjTOR mRNA was found in all developmental stages examined and exhibited the highest transcript level in cercariae, higher levels in eggs, 3-day-old and 35-day-old worms, and lower levels in 3-h-, 7-, 14- and 21-day schistosomula. Our data also showed that the transcript level in female adult worms is apparently higher than that in males (Fig. 2).

### Expression, purification and antigenicity analysis of rSjTOR-ed1

**Fig. 3:**
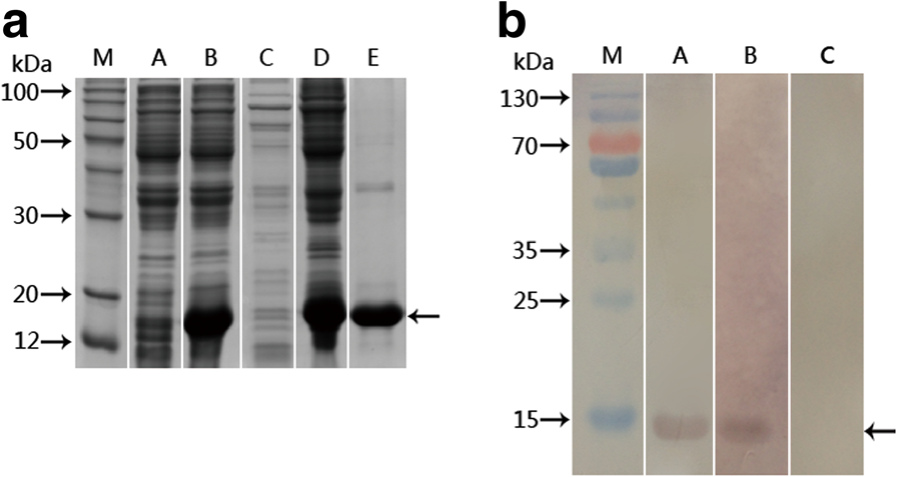
Analysis of expression and antigenicity of rSjTOR-ed1. **a** Expression and purification of SjTOR-ed1 in *E. coli*. Cell extracts and fractions from *E. coli* BL21 (*DE3*) transformed with pET28-SjTOR-ed1 were separated by 12% SDS-PAGE. Lanes A and B: total extract of a clone before and after induction with one mM IPTG; Lanes C and D: supernatant and inclusion bodies, respectively, after lysis; Lane E: rSjTOR-ed1 protein purified from inclusion bodies through Ni-NTA His-binding resin. **b** Western blot analysis of the antigenicity of rSjTOR-ed1. Lane M: molecular mass markers; Lanes A, B and C: purified rSjTOR-ed1 was probed with anti-rSjTOR-ed1 mouse serum (positive control), serum from mice infected with *S. japonicum* (experiment group), and normal mouse serum (negative control), respectively

The SjTOR-ed1 gene was inserted into the expression vector pET28a(+), and the recombinant protein with a molecular mass of ~14 kDa was successfully expressed in *E. coli* BL21 (*DE3*) when induced by IPTG (Fig. 3a). The rSjTOR-ed1 protein was purified using Ni-affinity chromatography under denaturing conditions and was refolded by dialysis against PBS that contained progressively decreasing concentrations of urea. The purity of the preparation was assessed using SDS-PAGE. The rSjTOR-ed1 was characterised further by Western blot. A positive band of ~14 kDa was observed when probed with *S. japonicum-*infected mouse serum and with anti-rSjTOR-ed1 mouse serum, but not in the naïve mouse serum, indicating that rSjTOR-ed1 had immunogenicity (Fig. 3b).

### Localisation of SjTOR protein in 35-day-old adult schistosome worms, cercariae and eggs

**Fig. 4:**
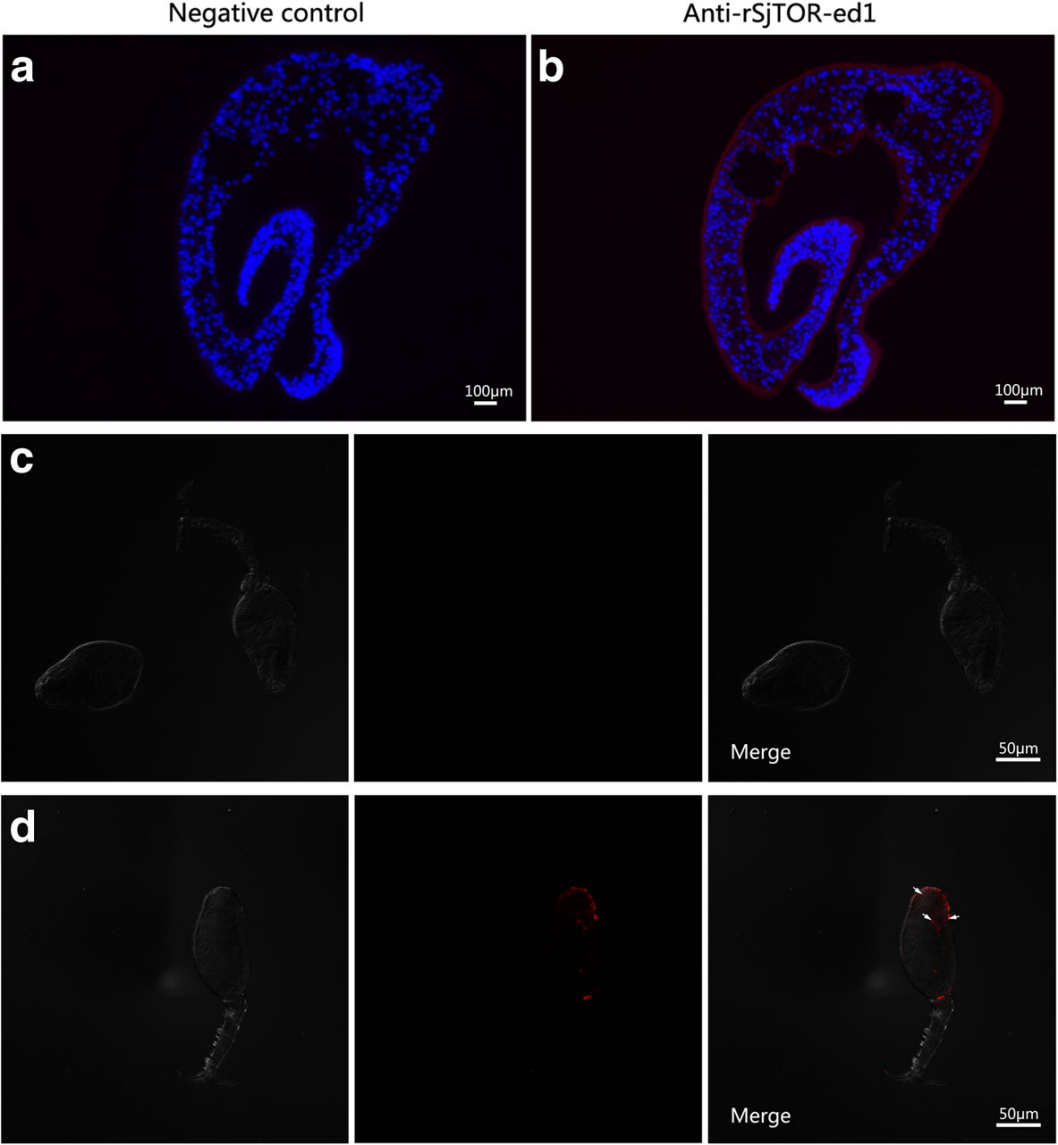
Localisation of SjTOR in 35-day-old *S. japonicum* and Cercariae. **a**-**d** Sections and cercariae were probed with serum from naive mice (**a** and **c**, negative control) or mice serum against rSjTOR-ed1 (**b** and **d**)

**Fig. 5:**
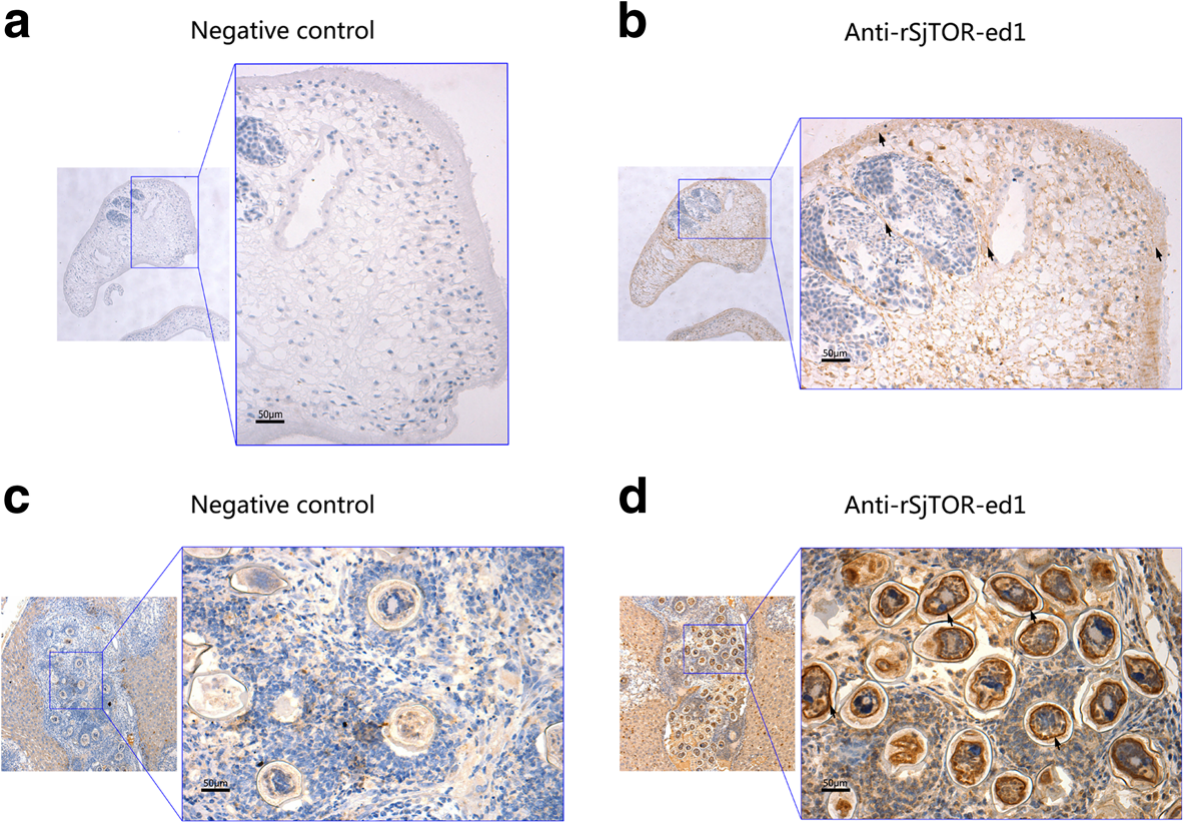
Immunohistochemical localisation of SjTOR in 35-day-old *S. japonicum* and Eggs. **a**-**d** The sections were detected by DAB, the positive staining of DAB is brown. Parasite slides were probed with serum from naive mice (**a**, negative control) or mice serum against rSjTOR-ed1 (**b**). The positive liver slides were probed with serum from naive mice (**c**, negative control) or mice serum against rSjTOR-ed1 (**d**)

Immunolocalization assays were performed to investigate the tissue localisation of SjTOR in adult schistosome worms, cercariae and eggs. The results revealed that native SjTOR was mainly distributed in the tegument, subtegumental musculature and at lower levels in the parenchyma of the adult parasites. Additionally, the obvious positive staining was also observed in the head of cercariae, and the tegument of miracidia matured in the eggs. No specific staining was found in the negative controls (Figs. 4 and 5).

### rSjTOR-ed1 could inhibit complement haemolysis and bind to C2

**Fig. 6:**
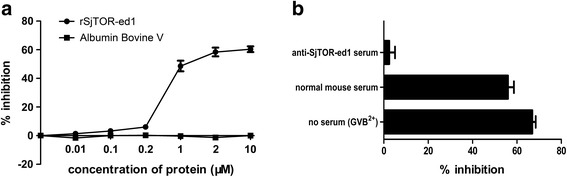
Haemolytic assays to determine the anticomplementary activity of rSjTOR-ed1. **a** The inhibition rate of hemolysis with increasing protein concentration. **b** The ability of pre-challenge anti-SjTOR-ed1 serum to inhibit the action of rSjTOR-ed1

**Fig. 7:**
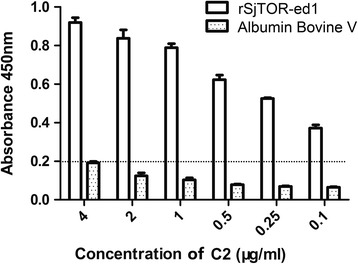
An ELISA assay for detecting the binding of rSjTOR-ed1 to C2. Plates were coated with rSjTOR-ed1 or Albumin Bovine V

The classic haemolytic assay was modified to evaluate the efficacy of rSjTOR-ed1 to inhibit complement activity. The results showed that the recombinant protein rSjTOR-ed1 could distinctly inhibit complement hemolysis with a dose-dependent pattern between 0.2–10 μM protein concentration, compared with albumin bovine V as a control protein. The hemolysis inhibition rate of rSjTOR-ed1 at a concentration of ~10 μM was up to ~60.26% (Fig. 6a). In addition, the anti-complement activity of rSjTOR-ed1 can be inhibited by pretreatment with anti-SjTOR-ed1 serum in the complement activation assay (Fig. 6b). Furthermore, an ELISA assay was developed to test C2 binding to rSjTOR-ed1, and the result indicated that rSjTOR-ed1 could bind C2 similarly to ShTOR-ed1 (Fig. 7).

### Protective efficacy induced by rSjTOR-ed1 in mice

To evaluate the protective effect induced by rSjTOR-ed1, the percent reductions in worm burden and liver egg numbers were calculated (Table 1). Mice immunized with rSjTOR-ed1 showed 24.51% (*t*_(12)_ = 1.78, *P* = 0.054) and 26.51% (*t*_(17)_ = 1.74, *P* = 0.002) reductions in worm burden, as well as 39.62% (*t*_(7)_ = 1.89, *P* = 0.028) and 32.92% (*t*_(14)_ = 1.76, *P* = 0.009) reductions in the egg count compared to the respective adjuvant control groups in two independent trials (In trial 1, five mice of the rSjTOR-ed1 group were dead as a result of ventilation installation failure and were not included in the analysis).

**Table 1:** Evaluation of the protective efficacy induced by rSjTOR-ed1

Group	Worm burden		Worm burden reduction in % (*P*-value)	EPG	EPG reduction in % (*P*-value)
	Mean ± SD	Range		Mean ± SD	
Trial 1					
PBS only (*n* = 10)	23.30 ± 5.68	17–35			
PBS + ISA206 (*n* = 10)	18.55 ± 8.14	9–31		29,384.56 ± 7956.68	
rSjTOR-ed1 (*n* = 5)	14.00 ± 2.00	12–16	24.51 (*P* = 0.054)	17,742.39 ± 9939.94	39.62 (*P* = 0.0284)
Trial 2					
PBS only (*n* = 10)	28.00 ± 4.44	16–31			
PBS + ISA206 (*n* = 10)	25.40 ± 4.50	19–32		32,848.89 ± 9912.66	
rSjTOR-ed1 (*n* = 10)	18.67 ± 4.24	12–25	26.51 (*P* = 0.002)	22,033.63 ± 7053.50	32.92 (*P* = 0.0092)

### Specific antibody analysis

**Fig. 8:**
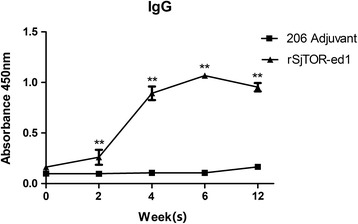
Kinetics of specific anti-rSjTOR-ed1 IgG induced in mice immunised with rSjTOR-ed1. Mice were injected with rSjTOR-ed1 and 206 adjuvant in PBS. Serum samples were obtained from each group at weeks 0, 2, 4, 6 and 12, and were analysed by ELISA. Asterisks indicate significant difference between the rSjTOR-ed1 immunised group and the 206 adjuvant control group (**P < 0.01)

Serum samples from different groups were assayed by ELISA to evaluate levels of specific anti-rSjTOR-ed1 IgG antibodies. After the first immunisation with rSjTOR-ed1, the amount of specific IgG antibodies increased significantly, and this antibody remained at a high level until the mice were euthanised. No significant differences in specific IgG levels were observed before and after vaccination in the adjuvant control group (Fig. 8). To evaluate IgG subtypes induced by rSjTOR-ed1 vaccination, levels of IgG1 and IgG2a antibodies specific to rSjTOR-ed1 were also detected by ELISA (Table 2). Levels of specific IgG1 increased rapidly after the first immunisation, whereas IgG2a levels increased gradually. Overall, the IgG1/IgG2a ratio peaked at week 4 and then decreased gradually, suggesting that vaccination with rSjTOR-ed1 could induce a Th1-type immune response. No significant differences in IgG1 and IgG2a levels were observed before and after vaccination in the adjuvant control group.

**Table 2:** The IgG1 and IgG2a immune profile induced by vaccination with rSjTOR-ed1 or 206 adjuvant

Week	IgG1		IgG2a		IgG1/IgG2a ratio
	ISA 206	rSjTOR-ed1	ISA 206	rSjTOR-ed1	
0	0.059 ± 0.001	0.058 ± 0.006	0.057 ± 0.001	0.050 ± 0.005	1.16
2	0.059 ± 0.003	0.156 ± 0.120*	0.057 ± 0.003	0.054 ± 0.002	2.89
4	0.064 ± 0.004	0.618 ± 0.190**	0.058 ± 0.001	0.082 ± 0.008*	7.54
6	0.065 ± 0.004	0.802 ± 0.019**	0.060 ± 0.002	0.124 ± 0.030**	6.47

*P < 0.05; **P < 0.01

## Discussion

The complement system is one of the first lines of innate immune defence against pathogen infection [31]. The sensitivity of parasites to complement is dependent on the life-cycle stage. Infectious cercariae are complement-sensitive, but become complement-resistant upon infection and undergo transformation to larval schistosomula while living within their host [32]. Additionally, parasites can use various strategies to evade the complement system. One is to prevent complement activation on the parasite surface [18, 19]. Thus, understanding the mechanisms involved in complement activation and resistance is not only important for the development of anti-*Schistosoma* vaccines [31] but also helps to reveal the mechanisms of schistosome immune evasion.

Bioinformatic analysis showed that SjTOR is a receptor with four transmembrane domains, including two predicted extracellular domains and has no signal peptide. The multi-membrane-spanning proteins probably depend on the hydrophobic transmembrane domains themselves to act as an internal signal sequence [33], or there are alternative functions related to the signalling capacity of the receptor within the cell [15]. Based on a secondary structure analysis, we predicted a surface-exposed extracellular domain 1 (ed1). The N-terminal extracellular domain 1 of ShTOR (ShTOR-ed1) as an isolated peptide has been shown to bind C2 [18]. Additionally, the C-terminal 11-amino-acids of the ShTOR-ed1 domain have high homology with a sequence in the C4b chain, which is responsible for binding to C2 [34], so it is necessary to test whether rSjTOR-ed1 could regulate complement activity. After a thorough review of the results in Fig. 1, we postulated that they might share a complement regulatory function, based on the high-level sequence identity of TORs and the H17 motifs. Although it is interesting to note several differences in the H17 motifs among the TORs, the same amino acids in the H17 motifs are also the same as the C4b chain [35]. In this study, our haemolytic assays and ELISA showed that rSjTOR-ed1 could inhibit classical pathway-mediated haemolysis of sheep erythrocytes in a dose-dependent manner by competing with C4b for binding to C2, similarly to the Sh-TOR-ed1 peptide and Sm-TOR-ed1 peptide [36]. Whether a peptide corresponding to the C-terminal 11-amino-acid of SjTOR-ed1 (SjTOR-H17) could more effectively regulate complement remains to be explored. However, the current speculation about the immune escape function of the schistosome TOR gene is based on the results of in vitro studies, and therefore more in-depth studies, particularly in vivo tests (e.g. RNAi), are needed to verify the role of the gene in schistosome development.

Quantitative real-time RT-PCR assays indicated that SjTOR transcripts were expressed in all stages tested. The highest expression was found in cercariae, where we detect it in the head of the cercaria, and the peak expression levels in cercariae have previously been described for SmTOR [10]. Cercariae are the first life-cycle stage to come into contact with the final host skin, so the impact of SjTOR on the fate of worms may be important at that specific time. Additionally, the high expression of SjTOR in eggs might occur because the eggs need to be protected *en route* to the environment ex vivo [15]. Female schistosomes at 42 days produce many eggs, and this might explain why SjTOR was expressed highly in this stage.

Many proteins on the tegument of the schistosome are key molecules for worms to survive in their host and are targets for the host immune attack. Previous research on tegument proteins also identified several molecules that successfully induced protective immune responses against schistosome infections, such as Sj23, Sm21.6, and Sm29 [35–37]. Using the antibody raised against rSjTOR-ed1, we found that SjTOR was mainly distributed in the tegument membrane of *S. japonicum* and also located in the head of the cercaria and the tegument of miracidia matured in the eggs, with the former supported by a previous proteomics study on tegumental surface proteins of *S. japonicum* in our laboratory [21]. Therefore, we speculated that SjTOR might be a good vaccine candidate antigen like SmTOR which is a tegument membrane protein and is expressed highly in cercariae [13]. Because of the important complement regulatory function of TOR-ed1 in schistosomes and its potential as a vaccine candidate against *S. mansoni*, the rSjTOR-ed1 protein was expressed, and the antigenicity of the recombinant protein analysed. Unlike rSmTOR-ed1, rSjTOR-ed1 does not form the dimerization and can be recognised by the serum from mice infected with *S. japonicum* [13]. We used rSjTOR-ed1 protein to immunise BALB/c mice to evaluate its protective efficacy against *S. japonicum* infection. A partial but significant reduction of worm burden and liver egg counts were induced in mice, and results obtained in the two experiments were similar. There is a large gap between the several immunoprotective effects induced by SjTOR and SmTOR, although both are significant, possibly due to the use of different adjuvants, or the difference between/in antigenicity of the recombinant antigen or *Schistosoma* species. The murine immune responses to rSjTOR-ed1 were investigated at the humoral level. ELISA assays showed that the purified rSjTOR-ed1 could elicit high levels of IgG antibodies until the mice were euthanised. IgG isotype analysis showed that rSjTOR-ed1 immunisation could induce both IgG1 and IgG2a antibodies, and the IgG1/IgG2a ratio peaked at week 4 and then decreased gradually, suggesting that vaccination with rSjTOR-ed1 may induce a Th1-type immune response.

## Conclusions

In summary, a gene encoding SjTOR was cloned and characterised. We confirmed that rSjTOR-ed1 could inhibit complement haemolysis and bind to C2. A partial but significant protection was obtained in mice vaccinated with rSjTOR-ed1.

## Abbreviations

DAPI: 2-(4-amidinophenyl)-6-indolecarbamidine dihydrochloride
ed1: Extracellular domain 1
ELISA: Enzyme-linked immunosorbent assay
EPG: Eggs per gram
HRP: Horseradish peroxidase
IPTG: Isopropyl-β-D-thiogalactopyranoside
PBS: Phosphate buffered saline
rSjTOR-ed1: Recombinant SjTOR-ed1
RT-PCR: Reverse transcription-ploymerase chain reaction
SD: Standard deviation
Sj: *Schistosoma japonicum*
SjTOR-ed1: First extracellular domain of the SjTOR
TOR: Tetraspanning orphan receptor
